# Parameter based 4D dose calculations for proton therapy

**DOI:** 10.1016/j.phro.2023.100473

**Published:** 2023-07-20

**Authors:** Franciska Lebbink, Silvia Stocchiero, Piero Fossati, Erik Engwall, Dietmar Georg, Markus Stock, Barbara Knäusl

**Affiliations:** a Medical University of Vienna, Department of Radiation Oncology, Vienna, Austria; b MedAustron Ion Therapy Centre, Wiener Neustadt, Austria; c Karl Landsteiner University of Health Sciences, Wiener Neustadt, Austria; d RaySearch Laboratories, Stockholm, Sweden

**Keywords:** 4D, Proton therapy, Dose calculations, Liver and pancreatic cancer, Interplay, Measurements, Pencil beam scanning

## Abstract

**Background and purpose** Retrospective log file-based analysis provides the actual dose delivered based on the patient’s breathing and the daily beam-delivery dynamics. To predict the motion sensitivity of the treatment plan on a patient-specific basis before treatment start a prospective tool is required. Such a parameter-based tool has been investigated with the aim to be used in clinical routine.

**Materials and Methods** 4D dose calculations (4DDC) were performed for seven cancer patients with small breathing motion treated with scanned pulsed proton beams. Validation of the parameter-based 4DDC (p-4DDC) method was performed with an anthropomorphic phantom and patient data employing measurements and a log file-based 4DDC tool. The dose volume histogram parameters ($D_{x\%}$) were investigated for the target and the organs at risk, compared to static and the file-based approach.

**Results** The difference between the measured and the p-4DDC dose was within the deviation of the measurements. The maximum deviation was 0.4Gy. For the planning target volume $D_{98\%}$ varied up to 15% compared to the static scenario, while the results from the log file and p-4DDC agreed within 2%. For the liver patients, $D_{33\%\mathrm{liver}}$ deviated up to 35% compared to static and 10% comparing the two 4DDC tools, while for the pancreas patients the $D_{1\%\mathrm{stomach}}$ varied up to 45% and 11%, respectively.

**Conclusion** The results showed that p-4DDC could be used prospectively. The next step will be the clinical implementation of the p-4DDC tool, which can support a decision to either adapt the treatment plan or apply motion mitigation strategies.

## Highlights

• Parameter-based 4D dose calculation can quantify the interplay effect for small movers.
• Inter-patient variability requires prospective 4D dose calculations in the clinic.
• Measured dose difference of 0.1 Gy with a moving phantom compared to 4DDC.
• Beam delivery dynamics did not influence the treatment robustness for motion <3 mm.

## 1 Introduction

Due to the sharp dose gradient, proton therapy can spare the healthy tissue better compared to conventional radiotherapy [1]. However, breathing motion, beam-delivery uncertainties and inter-fractional changes lead to degradation of target coverage and increase the dose to the surrounding tissues [2]. Four-dimensional dose calculation enables to simulate the interplay effect between breathing motion and the beam delivery dynamics, thereby the impact of motion on the treatment plan robustness during delivery. As this effect is patient and machine-specific, it is strongly recommended to perform 4D dose evaluations for each pathology on a patient-specific basis during the entire treatment course respecting the facility-specific beam delivery configuration [3], [4], [5]. For proton therapy several retrospective 4D dose calculation studies have been performed to quantify the impact of breathing motion [6], [7], [8], [9], [10], [11], [12]. Pakela et al. highlighted the importance of continuous plan evaluation based on repeated 4D computed tomography (4DCTs) during the treatment to assess the need for plan adaption [2]. In most studies, a log file-based approach was used, taking into account the information of every single fraction, reducing the interplay effect.

In earlier research, an anthropomorphic phantom was used to quantify the effect of the facility-specific configuration for synchrotron-based pencil beam scanning (PBS) proton therapy for motions in the thorax region [13]. Additionally, a log-file based 4D dose calculation framework (4D dose calculations (f-4DDC)) was validated and retrospectively employed to evaluate clinical data [14], [12]. This study converts this knowledge for clinical application with a prospective simplified 4D dose calculations (4DDC) tool implemented in the research version of a treatment planning system (TPS), further called parameter-based 4D dose calculations (p-4DDC). The advantage of the prospective p-4DDC is the capability to predict the effect of motion before the treatment starts, therefore providing support for clinical decisions and improving the clinical implementation of new treatment schemes.

The aim of this study was to validate p-4DDC based on patient data and measurements. The potential of p-4DDC, to assess the plan robustness of clinical liver and pancreas treatments, was investigated considering different parameters, i.e. breathing patterns and beam delivery dynamics, while the measurements aimed to verify the applicability for larger tumour motions.

## 2 Materials and Methods

### 2.1 4D dose calculations

A 4DDC framework recalculates the 3D dose distribution while taking into account the beam delivery dynamics and the breathing motion. The investigated prospective p-4DDC tool was available in the research version of RayStation (RS) 10A (v10.0.110) (RaySearch Laboratories AB, Sweden) based on fixed values for the beam delivery dynamics, namely dose rate (DR) in $10^{9}$ number of particles per second [GNP/s]; energy switching time (EST) [s]; scanning speed [cm/s]; breathing period (BP) [s]; 4DCT starting phase.

To retrieve the mean DR over all fractions the accelerator log files were post-processed for all delivered treatment plans, while the EST was a fixed value. It was assumed that the scanning speed was the same for all clinical treatment plans and the included measurements. The spots were recalculated on the different 4DCT phases after deformable image registration (DIR) was applied. The dose distribution resulting from the p-4DDC was accumulated according to the number of fractions the patient received.

**Fig. 1:**
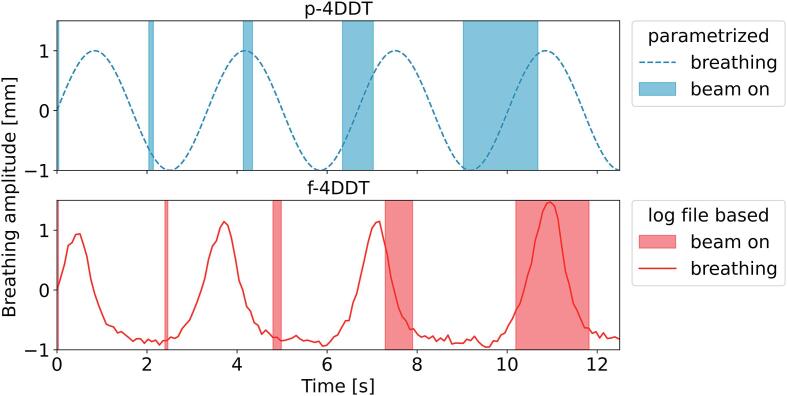
Illustration of the input from the two different 4DDC tools for patient liv3 plan1: (top) p-4DDC with a mean DR of 0.76 GNP/s, the fixed energy switching time of 2.0s and a fixed breathing period of 3.34s; (bottom) f-4DDC employing the breathing curve extracted from the surface scanner and the log file information to account for the varying accelerator time structure. The start and the end of each energy layer is displayed by the vertical bars.

**Fig. 2:**
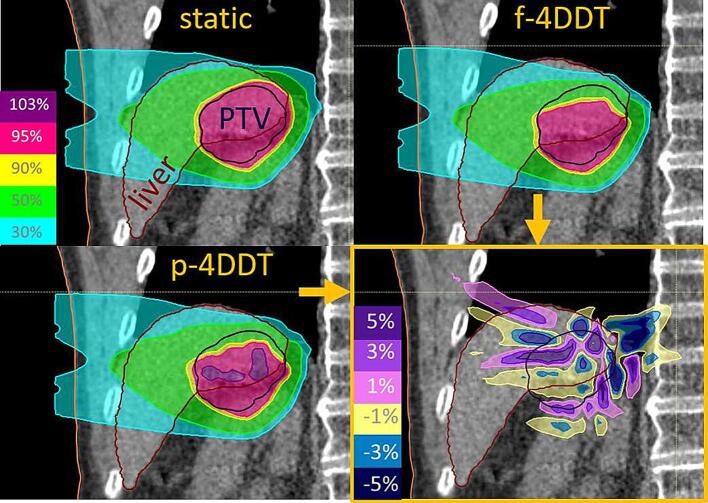
Dose distribution of static plan (upper left) and p-4DDC dose (lower left) with a mean DR of 0.76 GNP/s, the fixed energy switching time of 2.0s and a fixed breathing period of 3.34s, f-4DDC dose (upper right), dose difference between the two tools (lower right) of liv3 plan1.

Besides the p-4DDC, there was a log file-based tool (f-4DDC) employed for validation, which followed the same principle, as described above and illustrated in Fig. 1, but was based on the delivered time-resolved spot distribution and a varying breathing period over time as described previously [12]. An example of the static dose distributions recalculated with p-4DDC and f-4DDC is shown in Fig. 2. Breathing periods were extracted from the 4DCT or from the surface scanner signal during treatment if available.

### 2.2 Patient study

The p-4DDC was validated with patient data employing the results of the f-4DDC. Four pancreatic and three liver patients treated at the MedAustron Ion Therapy Centre (MedAustron) with proton therapy within the registry trial (trial number GS1-EK-4/350–2015) were included in this study, while only patients with small motion amplitudes were eligible for proton therapy at that time. Static computed tomography (CT) (Brilliance CT Big Bore Oncology, Philips, The Netherlands) and phase based 4DCT based on surface scanner (Sentinel, C-Rad, Sweden) were acquired as described previously [12]. 4DCT scans were acquired with eight breathing phases, all registered to the 0% breathing phase by DIR. The DIR between the different phases of the 4DCT were additionally used to extract the breathing amplitude at a point on the surface with the same cranio-caudal and left–right coordinates as the center of the plannning target volume (PTV) (Table 1).

**Table 1:** Patient plan characteristics: plan nomenclature, CT number, number of fractions, prescribed dose per fraction to the PTV; p-4DDC settings: mean DR [GNP/s], mean breathing period BP and mean breathing amplitude (BA). BP and BA were extracted from the surface scanner, if available, or from the 4DCT scan.

patients	CT	plans	No. of fx	dose/fx	DR (variation)	BP	BA
				[Gy]	[GNP/s]	[s]	[mm]
liv2	1	1	3	4.68	0.82 (2.2%)	3.8	0.7
		2	3		0.82 (4.0%)	3.9	1.5
		3	3		0.81 (2.2%)	3.8	1.2
	2	4	6		0.86 (10.1%)	4.3	1.6
liv3	1	1	3	4.68	0.76 (2.2%)	3.3	2.2
	2	2	7		0.78 (8.5%)	3.5	2.8
		3	2		0.82 (3.0%)	2.6	0.8
		4	3		0.82 (7.8%)	3.0	1.0
liv4	1	1	10	5.0	0.84 (9.3%)	3.8	1.1
pan2	1	1	6	4.0	0.52 (24.7%)	4.3	3.3
	2	2	4		0.68 (8.7%)	4.3	0.4
pan10	1	1	5	7.5	0.51 (11.1%)	4.4	2.3
pan11	1	1	5	8.0	0.82 (7.1%)	2.7	0.4
pan15	1	1	5	7.5	0.80 (6.4%)	4.5	0.6
ARDOS	1	1	9	2.0	0.45 (27.5%)	5.0	20.0
	1	2	4		0.16 (35.6%)		

All patients were treated with scanned pulsed quasi-discrete spot scanned proton beams delivered by a synchrotron. The prescribed dose varied between the different patients resulting in different biologically weighted mean dose to the PTV (based on a constant biological weighting factor of 1.1, Table 1). As the spot delivery was not synchronised with the breathing signal during irradiation, it was assumed that the irradiation started at the 0% breathing phase. All liver patients and one pancreatic (pan2) patient were treated with an EST of 2s, while the other pancreatic patients (pan10, 11, 15) were treated with 4s.

Clinical proton treatment plans were robustly optimised considering range and setup uncertainties with a PTV margin of 5mm as described previously [12]. Additional planning CT images with a different patient positioning were acquired to increase the beam incidence angles. This led to a total of 14 treatment plans on ten CTs from seven patients. 4D dose distributions of treatment plans based on the same CT were accumulated.

The influence of the 4DCT starting phase of the beam delivery (0%, 25%, 50%, 75%), DR (min, max and mean DR over all the fractions), breathing period (surface scanner and 4DCT) and energy switching time (1, 2 and 4 s) was investigated employing the p-4DDC.

### 2.3 Phantom study

To validate the p-4DDC tool dosimetrically, measurements were performed with the anthropomorphic Advanced Radiation DOSimetry phantom (ARDOS) [13]. Employed dosimeters for the time-resolved measurements were pin point (PP) chambers within the target (PP1-3,5) and in the surrounding tissue (PP4). A spread out Bragg peak (SOBP) treatment plan was created (RS7.99 Monte Carlo (MC) v4.3, RaySearch, Sweden) on the static CT to deliver a biologically-weighted dose of 2.0Gy(RBE) with two different dose rates. The 20 phases of the 4DCT were created by acquiring static CTs with the tumour shifted in 2mm steps.

Thirteen 4D measurements were performed on different days between March and December 2021 with a 2cm tumour motion and a breathing period of 5s, with a high (plan 1) or low (plan 2) DR (Table 1). The long time period between the measurements led to a higher variation for the measurements compared to the patient data. The tumour motion was synchronized with the beam delivery with a trigger signal, ensuring that every delivery started at the same motion phase and accounting for the delay between the trigger signal and the start of the beam. The mean DR of every measurement was extracted from the log files for the input of the p-4DDC tool (Table 1). Further, input for the p-4DDC was an energy switching time of 2s and a scanning speed of 300cm/s. For every measurement, the total dose of the PP chambers was compared to the respective p-4DDC dose values. In addition, the dose was extracted after the delivery of 20 energy layers.

### 2.4 Evaluation and statistics

Dose volume histogram (DVH) parameters ($D_{2\%},D_{98\%},D_{50\%},D_{1\mathrm{cc}}$ and $V_{95\%}$) were extracted for the target volumes, i.e. PTV and clinical target volume (CTV). The homogeneity index (HI) was defined as ($D_{2\%}$-$D_{98\%}$)/$D_{50\%}$ [15]. For the organs at risk (OARs), the $D_{33\%\mathrm{liver}}$ for the liver patients and the $D_{1\%\mathrm{stomach}}$ for the pancreas patients were considered. The different DVH parameters for the target and the OARs were reported by the median value and their corresponding ranges over all the treatment plans from the different patients.

For the validation measurements, the measured dose within the five PPs was compared to the average p-4DDC dose determined in a dedicated volume delineated on the planning CT.

A Wilcoxon test was performed (significance level p<0.05) to compare the different tools to each other and to compare the validation measurements with the average p-4DDC dose.

## 3 Results

### 3.1 Patient data

**Fig. 3:**
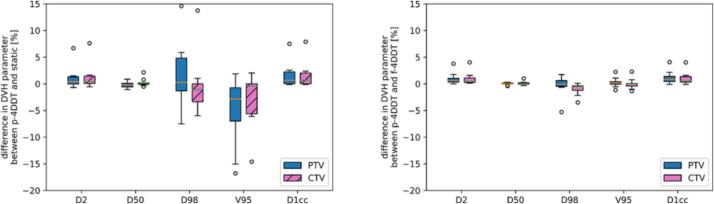
Difference in DVH parameters comparing p-4DDC using the DR, EST and breathing period given in Table 1 with static (left) and with the f-4DDC (right figure), where the boxplots show the median, the Q1 to Q3 quartile values of the data and the outliers.

Comparing the CTV DVH parameters of the p-4DDC dose to the static plan resulted in a median difference up to 1% for $D_{2\%},D_{50\%},D_{1\mathrm{cc}},D_{98\%}$ and $V_{95\%}$ (Fig. 3), while the median change for HI was 4.6% (-3.9% to 57.6%). For the PTV, the observed median difference was within 1% for all considered DVH parameters except for the $V_{95\%}$, which was −2.9% (-16.8% to 1.9%). Significant differences (p<0.05) were found for the $D_{2\%},D_{1\mathrm{cc}}$ and $V_{95\%}$ of the PTV and for $D_{2\%}$ and $D_{1\mathrm{cc}}$ of the CTV. For the OARs, a maximum change of up to 45% was found for $D_{1\%\mathrm{stomach}}$ for the pancreas patients and up to 35% for the $D_{33\%\mathrm{liver}}$ for liver patients, with a median change of 2.2% and 11.8%, respectively.

Comparing the p-4DDC against the f-4DDC tool revealed a median difference within 1% for $D_{2\%},D_{50\%},D_{98\%},V_{95\%}$ and $D_{1\mathrm{cc}}$ with outliers up to 5%, for CTV and PTV (shown in Fig. 3). The differences between the tools were statistically significant for the $D_{2\%}$ and $D_{1\mathrm{cc}}$ of the PTV and for $D_{2\%},D_{1\mathrm{cc}}$ and $D_{98\%}$ of the CTV. The small difference of the DVH parameters for the target showed that the motion itself had a larger impact than the 4DDC tool used for evaluation.

Comparing the two 4DDC tools, the HI varied more for the liver patients than for the pancreas patients, especially for the CTV, with a median change of 22.6% (4.5% to 63.2%) for the liver and 2.4% (0.2% to 3.2%) for the pancreas. For the OARs, a maximum difference of 10.7% was observed for $D_{1\%\mathrm{stomach}}$ for pancreas patients and 10.3% for $D_{33\%\mathrm{liver}}$ for liver patients comparing the two tools, with a median change averaged over all treatment plans of 1.4% and 1.5%, respectively.

The difference between static and p-4DDC was larger than between the two tools, reflected by the DVH parameters as well as the gamma analysis (Supplementary Material A), showing the applicability of the p-4DDC for prospective analysis of small movers. The variation of the beam delivery parameters, namely 4DCT starting phases, EST, DR and breathing periods showed no preferred accelerator settings for the selected indications and justified the parameters used for the p-4DDC (Supplementary Material B).

### 3.2 Phantom data

**Fig. 4:**
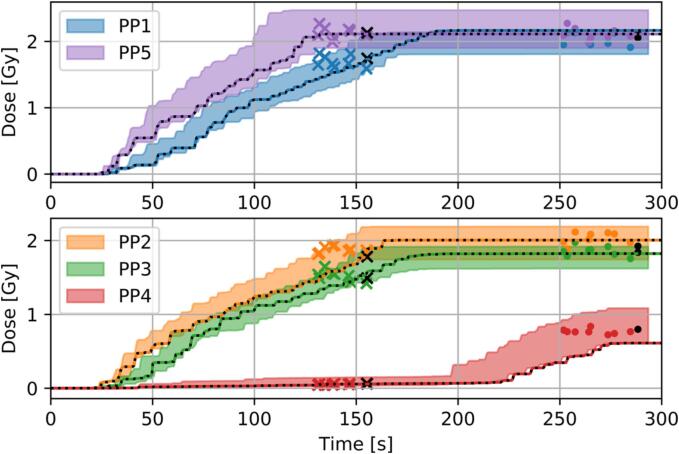
Measured dose over time for nine measurements with the ARDOS phantom with a 2.0cm tumour movement. The 4DDC dose calculations were computed for the different positions of the ionization chambers, where the 4DDC calculations were performed after 20 energy layers (marked as crosses) and at the end of the irradiation (marked as dots). The dose was measured time-resolved (0.5s interval) using PP ionization chambers. The black line and the black markers represent one scenario, with the measurement and the corresponding p-4DDC. The shaded area shows the variation between the measurements.

Calculations with p-4DDC for the treatment plan of the phantom showed a maximum deviation compared to static of 8%, 6.5%, 7.5%, 11.7% and 13.7% for PP1-5, respectively. The average of all scenarios calculated with p-4DDC agreed within 0.1Gy with the measurements for the 2cm tumour motion (Fig. 4) for all PP chambers. This variation was within the standard deviation of the measurements of 0.16Gy. The maximum dose difference between the measured and the corresponding p-4DDC dose for all thirteen scenarios was 0.4Gy for both time points (after 20 energy layers and at the end of irradiation) depending on the location of the PP chamber (shown in Supplementary Material Table C1). Only for PP3 a significant difference in dose was found between the measurements and the p-4DDC.

Measurements were performed with two different dose rates (Table 1), resulting in a variation of the median p-4DDC dose between these two groups of 5%. The measured median dose varied within 7% for PP1-3 and PP5 in the SOBP and up to 28% for PP4 in the penumbra region in the surrounding tissue. The mean DR in the patient study varied on average by 7.7% (maximum up to 24.7%), depending on the number of fractions applied, while for the ARDOS measurements, the DR varied between 0.39 GNP/s to 0.50 GNP/s for plan 1 and 0.14 GNP/s to 0.19 GNP/s for plan 2.

## 4 Discussion

In this study, p-4DDC was validated to prepare clinical implementation. It could be used prior to irradiation to identify the need of additional motion mitigation techniques, for prospective plan robustness analysis as a decision tool for plan adaptation, to support plan creation with respect to e.g. beam angle selection or definition of the start breathing phase, or for the analysis of new beam delivery dynamics and quality assurance (QA) purposes. After irradiation, f-4DDC could still be employed on the latest 4DCT to recalculate the dose as described previously [10], [12] in case of any unexpected motion patterns during irradiation or anatomical changes.

The agreement between p-4DDC and f-4DDC was excellent for the included patients, which were classified as small movers (maximum surface motion amplitude 3.3mm). Even the absolute dose difference caused by the interplay between tumour, organ and beam motion was small for this patient cohort, a strong inter-patient variability was observed, especially regarding the OARs. The higher variation between the liver patients could be caused by (the combination of) a lower median breathing period, higher median dose rate, lower EST, higher median breathing amplitude and different fractionation scheme compared to the pancreas patients.

All time-resolved measurements in the anthropomorphic ARDOS phantom showed a good agreement with the p-4DDC dose prediction. Those comparisons included a large tumour motion amplitude of 2.0cm, motivating the extension and clinical validation of the p-4DDC tool for large movers and other indications, like lung. Once more complex clinical cases would be considered, the dosimetric validation could be extended to motion patterns including lung and rib movement.

Still, the measurements and the validation with the patient data cannot be directly correlated, since the moving phantom simulated a lung patient, while our patient data includes liver and pancreas patients. Differences between the phantom and patient data included the larger breathing amplitude, the longer breathing period, DR variation and variation in the dose per fraction for the measurements. The DR, which could be set for the measurements, varied on a daily basis during patient treatment depending on the accelerator output. As the measurements were acquired over a period of one year the variation of the DR was essentially larger than for the clinical plans, which were irradiated in a time period of two weeks at maximum. The effect of dose rate variations was investigated with p-4DDC employing measurements and patient data, showing a median change of the $D_{50\%}$ within 1% for the patient data and within 2% for the phantom data in agreement with earlier results [13].

Several proton therapy centres, equipped with an anthropomorphic phantom and access to accelerator log files, performed studies to experimentally validate proton 4DDC including either multiple PP chamber measurements or 2D measurements [4], [8], [14], [16], [17], [18]. Those institutes without access to an anthropomorphic breathing phantoms and beam time, lack behind in performing a quantification of the interplay effect on a facility-specific basis [2], [3]. The presented p-4DDC tool could support this endeavour and help to quantify the impact on an individual basis being in line with the recommendation of a recent survey on the requirement of 4DDC [5].

The impact of variable breathing periods, the waveform and variation of the baseline position for larger breathing motions, showed that irregular and variable breathing periods have a larger influence on target inhomogeneity [4], [16], [19], [20]. This was in contrast to our results, where the fixed breathing periods for small amplitudes showed more hot spots for p-4DDC than for f-4DDC (Fig. 2).

Due to the small motion amplitudes of the included patients, the impact of the starting phase on the 4D dose distribution was limited, therefore, justifying the analysis of the data despite missing synchronisation with the beam delivery. However, for larger motion amplitudes, a synchronisation between beam delivery and breathing signal is crucial, especially for synchrotron facilities with larger inter-patient variations [5], [7]. Choosing the most suitable starting phase on a patient-specific basis is one of the fields where p-4DDC can support clinical implementation.

Anatomical changes can lead to a more pronounced dose degradation compared to the uncertainty of DIR [9].

Those aspects were not addressed in the frame of this study as the acquisition of the phase-based 4DCT scans on the same day as the planning CT in combination with the small motion amplitudes represents the ideal pre-condition for the DIR. Due to the small number of fractions, repeated CTs were scarce and not included in the evaluation. Anyhow, 4D dose calculation methods based on a single cycle 4DCT can lead to an underestimation of motion effects and are highly dependent on the acquired pre-treatment 4DCT [21], posing a limitation of this study. While adaptive treatment concepts based on repeated control imaging could reduce the inter-fractional variability, the incorporation of tumour tracking [22], [23] would reduce the intra-fraction variations.

For the small movers included in this study motion mitigation techniques including real-time tumour tracking would not be considered in a clinical treatment workflow. Going towards longer treatment schedules and larger motion amplitudes tumour tracking, repeated CT, daily control imaging as basis for synthetic CT creation including automatic contour delineation and deformable target contour propagation followed by automated replanning are essential [24], [25], [26], [27].

Further advancement of the study will be the clinical implementation of the parameter-based tool and the extension to carbon ions. The impact of different beam parameters has been investigated in this study and will be extended to new beam delivery parameters or new motion mitigation techniques, like gating tracking, more advanced rescanning methods, or the inclusion of novel robust optimisation strategies once envisaged for clinical implementation [7], [28], [29], [30].

To summarize, p-4DDC was validated employing measurements with an anthropomorphic phantom and clinical patient data. For small movers, the tool could be used to determine the impact of beam and organ motion for pancreas and liver cases in scanned proton therapy in a prospective setting. The impact of the different accelerator settings and fluctuations in the motion patterns were small. The measurements showed that p-4DDC can predict the interplay effect for large movers. The excellent agreement between p-4DDC with measurements and f-4DDC paves the way for prospective clinical application for a wide spread of indications.

## Funding

This research project was funded by the Science Call of the “Gesellschaft für Forschungsförderung, Niederösterreich m.b.H: SC19-013”.

## CRediT authorship contribution statement

**Franciska Lebbink:** Conceptualization, Data curation, Formal analysis, Funding acquisition, Investigation, Methodology, Project administration, Software, Supervision, Validation, Visualization, Writing - original draft, Writing - review & editing. **Silvia Stocchiero:** Formal analysis, Investigation, Software, Validation, Writing - review & editing. **Piero Fossati:** Methodology, Resources, Writing - review & editing. **Erik Engwall:** Conceptualization, Software, Writing - review & editing. **Dietmar Georg:** Conceptualization, Resources, Supervision, Writing - review & editing. **Markus Stock:** Conceptualization, Funding acquisition, Resources, Supervision, Writing - review & editing. **Barbara Knäusl:** Conceptualization, Formal analysis, Methodology, Project administration, Supervision, Validation, Writing - original draft, Writing - review & editing.

## Declaration of Competing Interest

The authors declare the following financial interests/personal relationships which may be considered as potential competing interests: E.E. reports a relationship with RaySearch Laboratories AB that includes employment.
