# Supplementary material for: Parameter based 4D dose calculations for proton therapy
Source: Phys Imaging Radiat Oncol. 2023 Jul 20;27:100473. doi: 10.1016/j.phro.2023.100473 (PMC10374597; doi:10.1016/j.phro.2023.100473)
Supplement: Supplementary data [file mmc1.pdf]

Supplementary material

A. Gamma analysis

Gamma pass rates were calculated in Python by comparing the dose distributions resulting from p-4DDC and f-4DDC as well as the p-4DDC dose distribution against static with a 2%/2 mm criteria, once considering all dose values above a lower dose cutoff of 20%, and once only in the PTV. For all patients, the gamma-pass rate comparing p-4DDC to static was 87.7% (73.2 % to 95.7 %) and 89.9% (55 % to 98.6 %), respectively, with one outlier (discussed in more detail below). Comparing the two tools with a 2%/2 mm criteria, the gamma pass ratio was 98.4% (92.0 % to 100 %) and 98.4% (70.7 % to 99.9 %), respectively. For the outlier, liv3 plan1 (dose distributions shown in Figure 2), the low gamma-pass rate can be explained by the low gamma-pass rate when comparing the p-4DDC tool with the

static distribution. This plan was irradiated three times, while plan 2-4 of the same patient was irradiated twelve times (Table 1).

B. Variation of delivery parameters

The different scenarios with varying 4DCT starting phases, EST, DR and breathing periods from the 4DCT were compared to the "reference" scenario (0% starting phase, mean DR, mean breathing period extracted from the surface scanner and the EST available during treatment, listed in Table 1). This revealed a median change within 1% for all considered DVH parameter ($D_{2\%}$, $D_{50\%}$, D_{1cc}, $D_{98\%}$ and $V_{95\%}$) of the CTV for pancreas and liver patients with an outlier up to 8.9% for $D_{98\%CTV}$ of liv3 (as shown in Figure B1). For this patient $V_{95\%}$ varied between −16.3 % to 3.6 %, while this was −5.8 % to 2.5 % for the other patients.

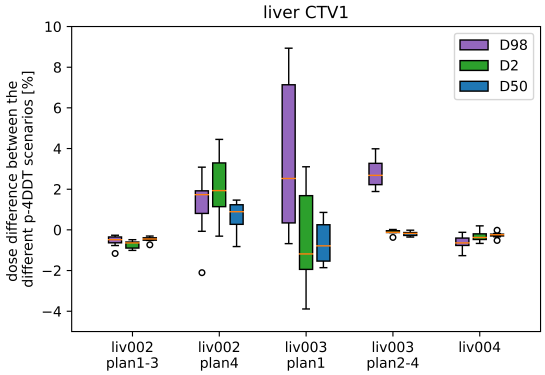

Figure B1: Difference in DVH parameters comparing the mean DR, EST of 2 s, mean breathing period from the surface scanner and a breathing phase of 0% with the other p-4DDC scenarios with varying DR (min, max), varying ESTs, mean breathing periods from the 4DCT, and varying starting breathing phases (25, 50 and 75 %). The boxplots show the median, the Q1 to Q3 quartile values of the data and the outliers.

The difference of all considered DVH parameters between the scenarios was smaller for the pancreatic patients than for the liver patients.

Comparing the individual effects of the DR, EST, 4DCT starting phase and breathing period showed equal variation for the different scenarios, with the median difference of all considered DVH parameters within 1%. None of the investigated variations were statistically significant. Although the DR variation over all patients was substantial (0.25 GNP/s to 1.08 GNP/s), the influence was negligible.

C. Dose values of phantom data

	Dose [Gy]			
	Planned	Measured		p-4DDC
	static		2 cm motion	
1	1.98	1.99±0.01	1.97±0.10	2.02±0.08
2	2.00	1.96±0.01	1.99±0.12	2.03±0.07
3	1.98	1.96±0.01	1.78±0.10	1.86±0.10
4	0.75	0.85±0.17	0.87±0.17	0.78±0.03
5	2.00	2.07±0.01	2.10±0.16	2.08±0.09

Table C1: Average dose values and its related standard deviation (SD) for the different PP locations for all 13 measurements and the dose calculations with the p-4DDC tool based on the dose rate from the 13 measurements. The measured static dose varied within 0.5% over a time period of a year.
